# Synergistic Regulation of δ-MnO_2_ Cathode via Crystal Engineering and pH Buffering for Long-Cycle Aqueous Zinc-Ion Batteries

**DOI:** 10.3390/ma18194632

**Published:** 2025-10-08

**Authors:** Fan Zhang, Haotian Yu, Qiongyue Zhang, Yahao Wang, Haodong Ren, Huirong Liang, Jinrui Li, Yuanyuan Feng, Bin Zhao, Xiaogang Han

**Affiliations:** 1 School of Electric Power, Civil Engineering and Architecture, Shanxi University, Taiyuan 030006, China; fanzhang@sxu.edu.cn (F.Z.); 202223503038@email.sxu.edu.cn (H.Y.); 202424314047@email.sxu.edu.cn (Q.Z.); 202323504037@email.sxu.edu.cn (Y.W.); renhaodong1@sxu.edu.cn (H.R.); lianghui3@sxu.edu.cn (H.L.); lijinrui1@sxu.edu.cn (J.L.); 202223503008@email.sxu.edu.cn (Y.F.); 2 State Key Laboratory of Electrical Insulation and Power Equipment, School of Electrical Engineering, Xi’an Jiaotong University, Xi’an 710049, China

**Keywords:** aqueous zinc-ion batteries, manganese dioxide, crystal engineering, pH buffer, long-cycle stability

## Abstract

Aqueous zinc-ion batteries (ZIBs) have emerged as a promising candidate for large-scale energy storage due to their inherent safety, low cost, and environmental friendliness. However, manganese dioxide (MnO_2_)-based cathodes, which are widely studied for ZIBs owing to their high theoretical capacity and low cost, face severe capacity fading issues that hinder the commercialization of ZIBs. This performance degradation mainly stems from the weak van der Waals forces between MnO_2_ layers leading to structural collapse during repeated Zn^2+^ insertion and extraction; it is also exacerbated by irreversible Mn dissolution via Mn^3+^ disproportionation that depletes active materials, and further aggravated by dynamic electrolyte pH fluctuations promoting insulating zinc hydroxide sulfate (ZHS) formation to block ion diffusion channels. To address these interconnected challenges, in this study, a synergistic strategy was developed combining crystal engineering and pH buffer regulation. We synthesized three MnO_2_ polymorphs (α-, δ-, γ-MnO_2_), identified δ-MnO_2_ with flower-like microspheres as optimal, and introduced sodium dihydrogen phosphate (NaH_2_PO_4_) as a pH buffer (stabilizing pH at 2.8 ± 0.2). The modified electrolyte improved δ-MnO_2_ wettability (contact angle of 17.8° in NaH_2_PO_4_-modified electrolyte vs. 26.1° in base electrolyte) and reduced charge transfer resistance (*R*ct = 78.17 Ω), enabling the optimized cathode to retain 117.25 mAh g^−1^ (82.16% retention) after 2500 cycles at 1 A g^−1^. This work provides an effective strategy for stable MnO_2_-based ZIBs, promoting their application in renewable energy storage.

## 1. Introduction

Since their conceptualization in the early 2010s as a safer alternative to flammable lithium-ion batteries (LIBs) for grid-scale energy storage [1], aqueous zinc-ion batteries (ZIBs) have evolved into a promising candidate for integrating intermittent renewable energy sources (e.g., wind and solar). This advancement is driven by their intrinsic safety (non-flammable aqueous electrolytes), low cost (a zinc crustal abundance of 0.0075%, ~1000 times higher than lithium), and high theoretical capacity of Zn anodes (820 mAh g^−1^) [2,3]. As the global energy transition accelerates, the demand for high-performance, low-cost, and environmentally benign energy storage systems has further amplified the significance of advancing ZIB technology [1,4].

Among various cathode materials for ZIBs, manganese dioxide (MnO_2_) has stood out for over a decade due to its high theoretical capacity (308–616 mAh g^−1^), low cost (0.96–1.6 USD kg^−1^), and non-toxicity [5,6]; however, its practical application remains constrained by severe capacity fading during cycling—a long-standing bottleneck that has limited ZIBs from competing with mature LIBs in large-scale applications [7,8]. This capacity decay stems from three interconnected challenges: first, intrinsic low electronic conductivity (10^−5^–10^−7^ S cm^−1^) leading to sluggish kinetics [9]; second, the strong Coulomb interaction between Zn^2+^ and the Mn-O lattice causing structural distortion/collapse [10]; and third, dynamic electrolyte pH fluctuations accelerating Mn dissolution via Mn^3+^ disproportionation (2Mn^3+^ → Mn^2+^ + Mn^4+^) [11,12]. Notably, structural stability and Mn dissolution are closely tied to the crystal structure of MnO_2_, making crystal engineering a critical yet underexplored strategy in early research [13].

MnO_2_ exhibits rich polymorphism (α-, β-, δ-, γ-MnO_2_), each with distinct electrochemical behaviors [14,15]. Layered δ-MnO_2_ offers large interlayer spacing (≈0.7 nm) for Zn^2+^ diffusion [16,17], and Huang et al. reported its higher capacity than α-MnO_2_ due to faster ion transport [18]; yet, pure δ-MnO_2_ suffers from structural degradation after long cycles, as weak van der Waals forces between layers cannot resist ion insertion stress [19,20]. γ-MnO_2_ (mixed tunnel-layered structure) combines tunnel rigidity and layer flexibility but remains plagued by Mn dissolution [21,22], while α-MnO_2_’s rigid 2 × 2 tunnels restrict ion diffusion [17]. Thus, screening optimal MnO_2_ polymorphs and suppressing Mn dissolution have become urgent priorities [23,24].

To mitigate Mn dissolution, strategies such as cation doping (e.g., Ni^2+^, Cu^2+^) and MnO_2_@carbon composites have been explored, but they often sacrifice capacity or increase cost [25,26,27,28]. Electrolyte optimization (e.g., Mn^2+^ additives) alleviates Mn loss via “dissolution-redeposition” but fails to address pH drift [29,30]—a root cause of Mn^3+^ disproportionation that also promotes insulating zinc hydroxide sulfate (ZHS) formation [31,32]. In recent years, pH buffer additives (e.g., phosphates) have emerged as a promising solution: phosphates maintain proton concentration via the H_2_PO_4_^−^/HPO_4_^2−^ equilibrium [33,34], and Liu et al. demonstrated that potassium dihydrogen phosphate (KH_2_PO_4_) improves MnO_2_ stability by stabilizing pH [35]. Other buffers (e.g., citrate, acetate) exhibit inferior performance in suppressing Mn dissolution [36,37], and comparative studies further revealed that sodium dihydrogen phosphate (NaH_2_PO_4_) outperforms KH_2_PO_4_ due to its stronger proton buffering capacity [38].

Despite separate advances in MnO_2_ crystal engineering (e.g., selecting high-performance polymorphs [39]) and electrolyte optimization (e.g., using pH buffers to suppress Mn dissolution [33]), no systematic work has integrated these two strategies to simultaneously address MnO_2_ structural degradation and Mn dissolution—two interconnected bottlenecks that together limit ZIB long-cycle stability. Specifically, two critical knowledge gaps persist: (1) systematic studies on the synergistic effect of crystal structure selection (screening optimal polymorphs) and pH buffer regulation [40] (suppressing Mn dissolution) are lacking, as most works address these strategies in isolation [41]; and (2) the mechanism behind NaH_2_PO_4_’s superiority over other additives (e.g., KH_2_PO_4_, NaHSO_4_) in enhancing MnO_2_ stability remains unclear [42,43]. Recent insights further highlight that advancing ZIBs requires both optimizing the MnO_2_ crystal structure (e.g., the expansion of δ-MnO_2_’s reversible layer expansion [44,45]) and regulating the interfacial environment (e.g., phosphate-induced surface passivation [46]), yet their integration remains underexplored limiting the development of high-stability MnO_2_ cathodes [47,48]. As Chen et al. emphasized, such synergistic integration is key to overcoming MnO_2_-based ZIB limitations [49,50,51,52,53].

To fill these gaps, this work synthesizes three MnO_2_ polymorphs (α-MnO_2_, δ-MnO_2_, γ-MnO_2_) to identify the optimal crystal structure, and systematically investigates the synergistic effect of δ-MnO_2_’s flower-like microsphere morphology (which provides fast ion diffusion paths) and NaH_2_PO_4_’s pH buffering (which stabilizes the interfacial environment). Notably, NaH_2_PO_4_’s regulation mechanism is compared with KH_2_PO_4_ and sodium bisulfate (NaHSO_4_) for the first time. This study not only reveals how the MnO_2_ crystal structure influences ZIB performance but also provides new insights into electrolyte design—ultimately advancing the practical application of low-cost, stable MnO_2_-based ZIBs.

## 2. Materials and Methods

### 2.1. Materials

Manganese sulfate monohydrate (MnSO_4_·H_2_O, 99%), potassium permanganate (KMnO_4_, 99%), ammonium persulfate ((NH_4_)_2_S_2_O_8_, 99%), zinc sulfate heptahydrate (ZnSO_4_·7H_2_O, 99%), NaH_2_PO_4_ (99%), KH_2_PO_4_ (99%), and NaHSO_4_ (99%) were purchased from Sinopharm Chemical Reagent Co., Ltd., (Shanghai, China). Zinc foil (0.1 mm thickness, 99.99%) was obtained from Tianjin Zhongneng Lithium Industry Co., Ltd., (Tianjin, China). Conductive carbon black (Super-P) was supplied by Timcal (Bodio, Switzerland). N-methylpyrrolidone (NMP, 99%) and polyvinylidene fluoride (PVDF, Mw ≈ 534,000) were purchased from Aladdin Chemical Co., Ltd., (Shanghai, China). All chemicals were used without further purification. Deionized water (18.2 MΩ·cm) was used throughout the experiments.

### 2.2. Synthesis of MnO_2_ Polymorphs

α-MnO_2_: A total of 0.3803 g manganese(II) sulfate hydrate (MnSO_4_·H_2_O, 2 mmol) and 0.2374 g potassium permanganate (KMnO_4_, 1.5 mmol) were separately dissolved in 20 mL deionized water under magnetic stirring for 30 min. The KMnO_4_ solution was slowly added dropwise to the MnSO_4_ solution, resulting in a brown suspension. After continuous stirring for 1 h, the mixture was transferred to a 50 mL Teflon -lined stainless-steel autoclave and reacted at 160 °C for 12 h. After cooling to room temperature, the product was centrifuged (8000 rpm, 5 min), washed alternately with deionized water and ethanol three times, and then dried at 80 °C in a vacuum oven for 12 h [54].

For the δ-MnO_2_, 24 mL of 2 mM MnSO_4_·H_2_O solution and 40 mL of 12 mM KMnO_4_ solution were mixed under magnetic stirring for 30 min. The resulting suspension was transferred to a 100 mL Teflon-lined autoclave and reacted at 180 °C for 18 h. The product was washed and dried using the same procedure as α-MnO_2_ [55].

For the γ-MnO_2_, 25 mL of 0.2 M ammonium persulfate ((NH_4_)_2_S_2_O_8_) solution and 25 mL of 0.2 M MnSO_4_ solution were mixed and stirred at 80 °C for 6 h, forming a brown precipitate. The product was collected via centrifugation, washed, and dried as described above [56].

### 2.3. Electrode Preparation and Battery Assembly

The cathode slurry was prepared by mixing MnO_2_ (active material), Super-P carbon black (conductive agent), and polyvinylidene fluoride (PVDF, binder) at a mass ratio of 7:2:1 in N-methylphthalimide (NMP) solvent. The slurry was uniformly coated on a stainless steel mesh (current collector, pretreated with 1 M HCl for 30 min to remove surface oxide and enhance adhesion) using a doctor blade, dried at 80 °C in a vacuum oven for 12 h, and then pressed at 5 MPa for 30 s to ensure compactness. The electrode thickness was measured at 5 random points using a micrometer, with a uniformity deviation < 5% (target thickness: 20 ± 1 μm). The mass loading of active material was controlled at 1.2–1.5 mg cm^−2^.

CR2025-type coin cells were assembled in air with the prepared MnO_2_ cathode, Zn foil (diameter 12 mm, thickness 0.1 mm) as the anode, glass fiber (GF/A, Whatman, Canrd Technology Co., Ltd., Donguan, China) as the separator, and 100 μL of 2 M ZnSO_4_ + 0.5 M MnSO_4_ aqueous solution as the base electrolyte. For buffer additive experiments, 0.1 M NaH_2_PO_4_, KH_2_PO_4_, or NaHSO_4_ was added to the base electrolyte (100 μL per cell), respectively.

### 2.4. Material Characterization

X-ray diffraction (XRD) patterns were recorded on a Bruker D8 Advance diffractometer (Bruker (Beijing) Scientific Technology Co., Ltd., Beijing, China) with Cu Kα radiation (λ = 1.5418 Å) at 40 kV and 40 mA, scanning from 10° to 80° with a step size of 0.01°. To ensure the reliability of subsequent crystallite size calculations via the Scherrer equation, instrumental broadening (*β*_instr_) was first calibrated using a silicon standard (JCPDS #00-002-1235), which was measured under identical experimental conditions (40 kV, 40 mA) as the MnO_2_ samples—this step is critical for isolating size-induced peak broadening from experimental artifacts. For crystallite size determination, the full width at half maximum (*β*, FWHM) of each MnO_2_ diffraction peak was converted to radians. The corrected FWHM (*β*_corrected_) was derived by quadrature subtraction of *β*_instr_ from the measured FWHM (*β*_measured_) using Equation (1). This calculation ensures only broadening induced by crystallite size is included in the Scherrer analysis, eliminating interference from instrumental factors.

(1) $$\beta_{corrected}=\sqrt{\beta_{measured}^{2}-\beta_{instr}^{2}}$$

Brunauer–Emmett–Teller (BET) specific surface area and pore structure analyses of δ-MnO_2_ were performed via nitrogen adsorption desorption isotherms at 77 K using a Micromeritics TriStar II 3020 analyzer (Micromeritics Instrument (Shanghai) Ltd., Shanghai, China). Prior to testing, the samples were degassed at 150 °C for 6 h under vacuum to eliminate adsorbed water and surface impurities.

Scanning electron microscopy (SEM) and energy-dispersive X-ray spectroscopy (EDS) were performed using a Hitachi SU8010 microscope (Hitachi High-Tech Scientific Solutions (Beijing) Co., Ltd., Beijing, China) at an accelerating voltage of 15 kV.

X-ray photoelectron spectroscopy (XPS) was conducted on a Thermo ESCALAB Xi+ spectrometer (Thermo Fisher Scientific (China) Co., Ltd., Shanghai, China) with Al Kα excitation (1486.6 eV), and the binding energies were calibrated using the C 1s peak at 284.8 eV. Contact angle measurements were carried out using a Kruss DSA100 instrument (Krüss Scientific Instruments (Shanghai) Co., Ltd., Shanghai, China) with 5 μL electrolyte droplets.

### 2.5. Electrochemical Measurements

Cyclic voltammetry (CV) and electrochemical impedance spectroscopy (EIS) were performed on a Bio-Logic SP200 electrochemical workstation (Bio-Logic (China) Co., Ltd., Hongkong, China). CV curves were recorded at scan rates of 0.5, 1.0, and 2.0 mV s^−1^ in the voltage range of 0.8–1.8 V (vs. Zn^2+^/Zn). EIS measurements were conducted at open circuit voltage with a frequency range of 100 kHz to 100 mHz and an AC amplitude of 5 mV. Galvanostatic charge–discharge (GCD) tests were performed on a LAND CT2001A battery tester (Wuhan LAND, Wuhan, China) at current densities of 0.2–5 A g^−1^ within the voltage window of 1.0–1.8 V (vs. Zn^2+^/Zn). Galvanostatic intermittent titration technique (GITT) tests were conducted on a three-electrode coin cell (with Zn wire as the reference electrode) by charging/discharging at 0.1 A g^−1^ for 10 min, followed by a 120 min rest period to restore the steady state. All tests were conducted at room temperature (25 ± 2 °C).

#### 2.5.1. Calculation of CV-Derived *b*-Values

To clarify the charge storage mechanism (diffusion-controlled vs. capacitive), the relationship between peak current (*i*) and scan rate (*ν*) was analyzed using the empirical power-law equation [23,57]:

(2) $$I={a\nu }^{b}$$

where *a* and *b* are constants. Taking the logarithm of both sides converts the equation to a linear form:

(3) log(i)=b log(ν)+log(a)

The *b*-value is obtained from the slope of the *log*(*i*) vs. *log*(*v*) plot. A *b*-value of ~0.5 indicates a diffusion-controlled process (dominated by ion insertion/extraction), while a *b*-value of ~1.0 indicates a capacitive-controlled process (dominated by surface redox reactions). For each redox peak in the CV curves, peak currents at different scan rates (0.5, 1.0, 2.0 mV s^−1^) were extracted, and *log*(*i*) − *log*(*v*) plots were constructed to calculate the *b*-values.

#### 2.5.2. Calculation of GITT-Derived Ion Diffusion Coefficients

The Zn^2+^ diffusion coefficient ($D_{Zn^{2+}}$) was calculated using the following equation [25,28], derived from Fick’s second law for semi-infinite diffusion:

(4) $$D_{Zn^{2+}}=\frac{4}{\pi \tau }{\left(\frac{n_{m}V_{m}}{SF\Delta E_{s}}\right)}^{2}{\left(\frac{\Delta E_{t}}{\Delta E_{s}}\right)}^{2}$$

where the following definitions are used:

*τ* = duration of current pulse (s);

*n*_m_ = moles of active material (mol);

*V*_m_ = molar volume of δ-MnO_2_ (17.3 cm^3^ mol^−1^, calculated from its density of 5.03 g cm^−3^);

*S* = electrode/electrolyte contact area (cm^2^);

*F* = Faraday’s constant (96,485 C mol^−1^);

Δ*E*_s_ = steady-state voltage change after pulse (V);

Δ*E*_t_ = total voltage change during pulse (V, excluding IR drop).

#### 2.5.3. Calculation of Capacitive and Diffusion-Controlled Contributions

To quantitatively separate the capacitive and diffusion-controlled contributions to charge storage, the standard Dunn method was applied to CV data at scan rates of 0.5, 1.0, and 2.0 mV s^−1^ [23,57]. The total current (*i*) at a given voltage is decomposed into two components using the following equation:

(5) $$i=k_{1}\upsilon +k_{2}\upsilon^{1/2}$$

where the following definitions are used:

*k*_1_*υ* = capacitive contribution (surface redox reactions);

*k*_2_*υ*^1/2^ = diffusion-controlled contribution (Zn^2+^/H^+^ insertion/extraction);

υ = scan rate (V s^−1^);

*k*_1_ and *k*_2_ are fitting constants.

For each redox peak in the CV curves, the values of *k*_1_ (slope) and *k*_2_ (intercept) were obtained by plotting *i*/*υ*^1/2^ against *υ*^1/2^ and performing linear fitting. The capacitive contribution ratio was further quantified as the percentage of the area under the k_1_ curve relative to the total CV peak area at a given scan rate.

## 3. Results and Discussion

### 3.1. Crystal Structure and Morphology of MnO_2_ Polymorphs

**Figure 1:**
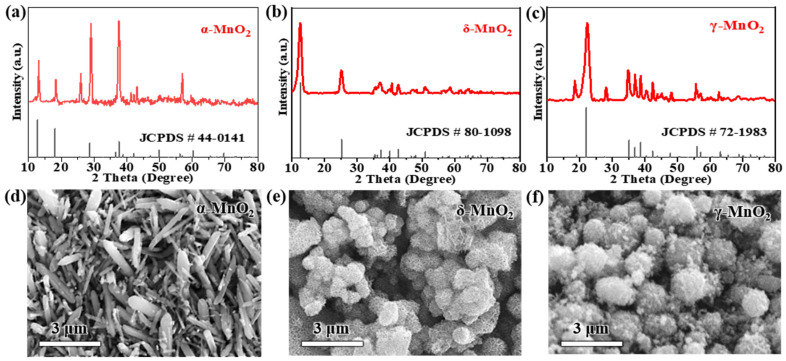
XRD patterns of (**a**) α-MnO_2_, (**b**) δ-MnO_2_, and (**c**) γ-MnO_2_; SEM images of (**d**) α-MnO_2_ (rod-like structure), (**e**) δ-MnO_2_ (flower-like microspheres), and (**f**) γ-MnO_2_ (spherical aggregates).

To confirm the phase purity and crystal symmetry of the synthesized MnO_2_ polymorphs, XRD characterization was first performed. As presented in Figure 1a, α-MnO_2_ exhibits well-resolved characteristic diffraction peaks at 2*θ* = 12.7° (110 reflection), 18.0° (200 reflection), 28.6° (310 reflection), 37.5° (211 reflection), 41.9° (301 reflection), 49.9° (411 reflection), and 60.2° (521 reflection), which are in perfect agreement with the standard tetragonal α-MnO_2_ phase (JCPDS #44-0141) [54]. Notably, the (310) peak of our synthesized α-MnO_2_ appears at 28.7°, showing a 0.1° positive shift relative to the reference 28.6° in JCPDS #44-0141 [54]. This shift arises from K^+^ intercalation from the KMnO_4_ precursor during hydrothermal synthesis: K^+^ ions enter the 2 × 2 tunnels of α-MnO_2_ to balance lattice charge, inducing mild lattice compression that reduces the d-spacing of the (310) plane (from 3.11 Å in the reference to 3.10 Å in our sample) [14]. Per Bragg’s law (nλ = 2dsin*θ*), the decreased d-spacing directly causes the peak to shift to higher 2*θ* values, which is consistent with observations of K^+^-induced lattice distortion in hydrothermally synthesized α-MnO_2_ [30]. The sharpness and intensity of these peaks confirm the formation of a highly crystallized 2 × 2 tunnel structure, which is attributed to the oriented crystal growth induced by the 160 °C hydrothermal treatment—high temperature promotes the ordered arrangement of Mn-O octahedra into rigid tunnel frameworks.

For δ-MnO_2_ (Figure 1b), the XRD pattern displays diffraction peaks at 2*θ* = 12.2° (001 reflection), 24.7° (002 reflection), 36.8° (11-1 reflection), and 65.0° (004 reflection), which match the layered birnessite structure (JCPDS #80-1098) [55]. Notably, the (001) peak is both broad and intense: the broadness is associated with the ultrathin nature of the layered nanosheets, while the high intensity indicates a high degree of in-plane layer ordering. Quantitative analysis of this XRD pattern further confirms δ-MnO_2_’s layered structure: using Bragg’s law (*n*λ = 2dsin*θ*) with *n* = 1, λ = 1.5418 Å (Cu Kα radiation), and sin(12.2°) ≈ 0.211, the interlayer spacing from the (001) peak is calculated as ~3.63 Å. This value is sufficient to accommodate hydrated Zn^2+^ (effective ionic diameter ≈ 4.3 Å), directly supporting the structural feasibility of reversible Zn^2+^ insertion/extraction. To characterize the nanocrystalline nature of δ-MnO_2_, the crystallite size was calculated via the Scherrer equation ($D=\frac{K\lambda }{\beta \cos\theta }$) with strict unit conversion and methodological rigor: K = 0.9 (shape factor for plate-like nanosheets [8,58]), λ = 1.5406 Å (0.15406 nm; Cu Kα_1_) (adopting the dominant Kα_1_ component, a standard choice for Scherrer crystallite size analysis), *β* = 0.8° (FWHM of (001) peak, converted to 0.01396 rad to meet the equation’s unit requirement), and *θ* = 6.1° (converted to 0.1065 rad). It is noted that the composite Cu Kα value (1.5418 Å) was used for Bragg’s law-based interlayer spacing calculation—this convention is common for d-spacing estimation as it reflects the full Cu Kα emission profile, and both wavelength choices (1.5418 Å for Bragg, 1.5406 Å for Scherrer) align with standard XRD practices, ensuring internal consistency in structural characterization.

Prior to final crystallite size calculation, instrumental broadening was corrected using a silicon standard (JCPDS #00-002-1235) with *β*_instr_ = 0.3°; this step isolates the broadening induced by crystallite size from experimental artifacts, a critical prerequisite for reliable Scherrer analysis. After correction, the *β*_corrected_ values for δ-MnO_2_ range from 0.0129 to 0.0204 rad, yielding crystallite sizes of 9.8–10.5 nm (average: 10.2 ± 0.5 nm) (Table S1). This size range is not only equivalent to ~34 Mn-O octahedral layers (each ~0.3 nm thick [6])—consistent with the moderate peak narrowing in Figure 1b (indicative of nanoscale crystallinity) and literature [55,58], but also falls squarely within the Scherrer equation’s applicability window for nanocrystalline materials, where the method reliably provides first-order crystallite size estimates [33,43]. This well-ordered layered structure is particularly favorable for Zn^2+^ intercalation, as it provides sufficient interlayer spacing (≈0.7 nm) to accommodate the reversible insertion/extraction of Zn^2+^ without severe lattice distortion.

As for γ-MnO_2_ (Figure 1c), its XRD pattern shows mixed diffraction features of α-MnO_2_ and β-MnO_2_, with key peaks at 2*θ* = 12.8° (110 reflection), 25.7° (220 reflection), 37.3° (310 reflection), and 42.0° (211 reflection)—consistent with the mixed tunnel-layered structure of the standard γ-MnO_2_ phase (JCPDS #72-1983) [56]. These peaks align with the mixed-phase literature [16,22], where the 12.8° peak is attributed to α-MnO_2_-like tunnel domains and the 25.7° peak to β-MnO_2_-like compact domains; notably, γ-MnO_2_’s electrochemical performance (211.5 mAh g^−1^ at 0.2 A g^−1^ and 48.3% capacity retention after 1000 cycles, detailed in Section 3.2) confirms its domain distribution consistency with reported γ-MnO_2_ samples [10,28]. Two distinct peak shifts are observed relative to the reference: the (220) peak shifts from 25.7° to 25.9°, and the (310) peak shifts from 37.3° to 37.5°, both by 0.2°. These shifts stem from two factors tied to the 80 °C heating coprecipitation synthesis: first, the relatively low temperature leads to incomplete crystallization, resulting in a small crystallite size (~18 nm, calculated via the Scherrer equation) that introduces lattice microstrain—this strain compresses the unit cell and shifts peaks to higher 2*θ* values [16]. Second, trace SO_4_^2−^ ions from the (NH_4_)_2_S_2_O_8_ precursor adsorb onto the surface of γ-MnO_2_, creating localized electrostatic interactions that further compress the mixed tunnel-layered lattice [17]. This mixed-phase characteristic originates from incomplete phase transition during the 80 °C heating coprecipitation process: the relatively low reaction temperature inhibits the full crystallization of a single phase, leading to the coexistence of α-MnO_2_-like tunnel domains and β-MnO_2_-like compact domains [57].

SEM images further reveal distinct morphologies of the three MnO_2_ polymorphs, which are tightly correlated with their synthesis methods and intrinsic crystal structures (Figure 1d–f). α-MnO_2_ forms one-dimensional rod-like structures with lengths of 2–5 μm and diameters of 200–500 nm. This rigid, elongated morphology is a direct consequence of the anisotropic growth of its tetragonal tunnel structure under high-temperature hydrothermal conditions [59]; this rigid, elongated morphology (aspect ratio 4–25) may increase electrolyte tortuosity—consistent with literature showing that high-aspect-ratio 1D materials disrupt electrolyte flow and elevate diffusion resistance [29]—which could limit ion diffusion under high current densities (further validated by EIS analysis in Section 3.3), potentially limiting ion diffusion under high current densities.

In contrast, δ-MnO_2_ exhibits hierarchical ‘flower-like microspheres’—spherical structures (200–500 nm in diameter) assembled from radially oriented ultrathin nanosheets (~5 nm thick)—a morphology that provides large specific surface area and short ion diffusion paths, consistent with reported flower-like δ-MnO_2_ [55,58]. This unique morphology is formed via 180 °C hydrothermal self-assembly: the high temperature drives the nucleation of MnO_2_ nanosheets, which further aggregate into microspheres to minimize surface energy. Importantly, this hierarchical structure provides two key advantages for electrochemical performance: it delivers a large specific surface area (63.05 m^2^ g^−1^) to expose abundant active sites; it creates inter-nanosheet gaps (≈50 nm) that enhance electrolyte penetration; and it shortens ion diffusion paths to <100 nm—both critical for accelerating reaction kinetics [60]. This claim is validated by three lines of evidence: (1) the 200–500 nm diameter of flower-like microspheres limits radial diffusion paths to <250 nm; (2) the ultrathin nanosheet building blocks (≈5 nm) further shorten ion transport distances [58]—with quantitative confirmation of fast Zn^2+^ diffusion provided by GITT analysis in Section 3.5; and (3) the ultrathin nanosheet building blocks (≈5 nm) further shorten ion transport distances. BET analysis further quantifies the textural differences between polymorphs (Figure S1): δ-MnO_2_ exhibits a specific surface area (SSA) of 63.05 m^2^ g^−1^, total pore volume of 0.21 cm^3^ g^−1^, and mesoporous size distribution (peak at ~15 nm); α-MnO_2_ (28.3 m^2^ g^−1^, 0.08 cm^3^ g^−1^, macropores > 50 nm) and γ-MnO_2_ (38.2 m^2^ g^−1^, 0.12 cm^3^ g^−1^, mixed meso/macropores) show lower porosity. This high mesoporosity of δ-MnO_2_ enhances electrolyte penetration and active site accessibility, directly contributing to its superior electrochemical performance described in Section 3.2 [58].

γ-MnO_2_, synthesized via low-temperature heating coprecipitation, consists of spherical aggregates with diameters of 1–3 μm, and the surface of these aggregates is covered with small secondary particles (≈100 nm). The rapid nucleation and growth at 80 °C lead to the formation of this aggregated morphology: fast nucleation produces numerous small primary particles, which immediately aggregate into spheres to reduce surface energy. While the spherical shape partially increases active site exposure, the dense aggregation of secondary particles limits electrolyte penetration—consistent with lower specific surface areas for α-MnO_2_ (28.3 m^2^ g^−1^) and γ-MnO_2_ (38.2 m^2^ g^−1^) compared to δ-MnO_2_. This compact morphology of γ-MnO_2_ may also lead to higher charge transfer resistance, as it restricts the contact between the active sites and electrolyte [61].

### 3.2. Electrochemical Performance of MnO_2_ Polymorphs

**Figure 2:**
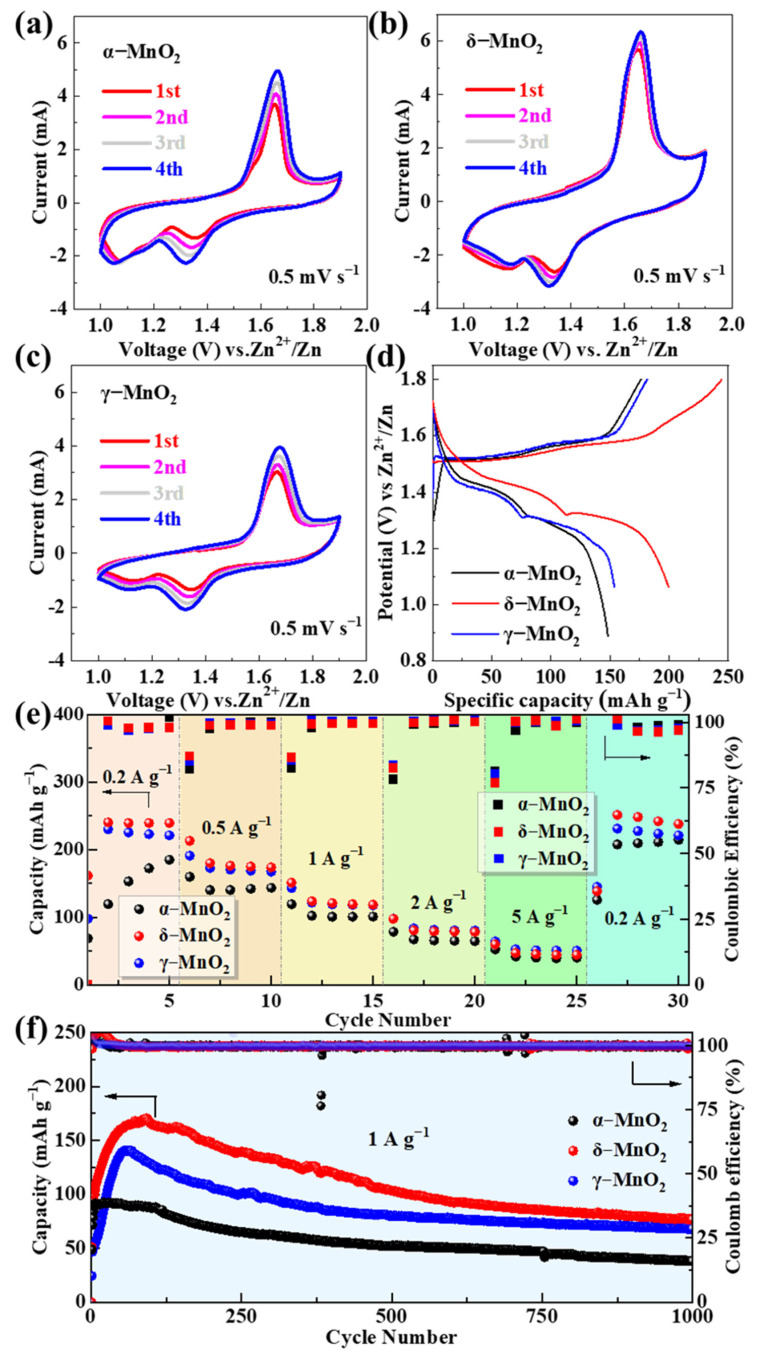
CV curves of (**a**) α-MnO_2_, (**b**) δ-MnO_2_, and (**c**) γ-MnO_2_ at 0.5 mV s^−1^ for the first four cycles; (**d**) GCD curves at 0.2 A g^−1^; (**e**) rate performance at current densities of 0.2–5 A g^−1^; (**f**) long-cycle stability at 1 A g^−1^.

The electrochemical behaviors of the three MnO_2_ polymorphs were evaluated using CV, GCD, and EIS to establish their structure–performance relationships. All tests in this section were conducted in a buffer-free base electrolyte (2 M ZnSO_4_ + 0.5 M MnSO_4_) to establish a baseline for evaluating the intrinsic performance of MnO_2_ polymorphs, with the goal of identifying the optimal candidate for subsequent pH buffer modification. Figure 2a–c shows the CV curves of α-MnO_2_, δ-MnO_2_, and γ-MnO_2_ at a scan rate of 0.5 mV s^−1^ for the first four cycles. All samples exhibit two pairs of well-defined redox peaks, which are characteristic of the reversible insertion/extraction of Zn^2+^ and H^+^ in MnO_2_-based cathodes for aqueous ZIBs [62]. The oxidation peak at ~1.65 V corresponds to the deintercalation of Zn^2+^/H^+^ from the MnO_2_ lattice, while the two reduction peaks at ~1.33 V and ~1.10 V are attributed to the stepwise insertion of Zn^2+^/H^+^—a result of the sequential interaction between Zn^2+^/H^+^ and the Mn-O framework [63]. Among the three polymorphs, δ-MnO_2_ displays the largest redox peak area and highest peak current, indicating the highest electrochemical activity. To quantify this superiority, we integrated the first-cycle CV reduction peaks (1.33 V/1.10 V) at 0.2 mV s^−1^ using the Bio-Logic SP200 workstation’s built-in function: δ-MnO_2_ exhibits a total reduction charge of 138.6 mC cm^−2^, which is 28.3% higher than γ-MnO_2_ (108.0 mC cm^−2^) and 45.1% higher than α-MnO_2_ (95.5 mC cm^−2^). This aligns with previous literature linking CV peak charge to active site utilization [8] and further confirms δ-MnO_2_’s enhanced electrochemical activity.

This superiority originates from its flower-like microsphere structure, which provides a large specific surface area (63.05 m^2^ g^−1^, confirmed by BET) to expose more active sites and shortens ion diffusion paths (<100 nm) to accelerate reaction kinetics. Additionally, the CV curves of δ-MnO_2_ show the highest overlap between the first and fourth cycles, reflecting superior reaction reversibility compared to α-MnO_2_ and γ-MnO_2_—this is critical for maintaining stable capacity during long cycles.

The GCD curves of the three polymorphs at 0.2 A g^−1^ (Figure 2d) exhibit distinct voltage plateaus that correspond to the redox peaks in the CV curves, further verifying the consistency of the electrochemical reaction mechanism. δ-MnO_2_ delivers an initial discharge capacity of 244.4 mAh g^−1^, which is higher than that of γ-MnO_2_ (211.5 mAh g^−1^) and α-MnO_2_ (187.3 mAh g^−1^). The longer voltage plateau of δ-MnO_2_ (1.2–1.4 V, accounting for ~60% of the total discharge time) indicates a more efficient ion storage process, as the layered structure of δ-MnO_2_ allows for uniform Zn^2+^/H^+^ insertion without significant lattice distortion [55]. In contrast, α-MnO_2_ shows a shorter plateau and lower capacity due to its rigid 2 × 2 tunnel structure, which restricts ion diffusion under low current densities, while γ-MnO_2_’s aggregated spherical morphology leads to partial active site inaccessibility, resulting in moderate capacity.

The rate performance of the three polymorphs was tested at current densities ranging from 0.2 to 5 A g^−1^ (Figure 2e). When the current density returns to 0.2 A g^−1^ after cycling at 5 A g^−1^, δ-MnO_2_ retains a discharge capacity of 271.6 mAh g^−1^—higher than γ-MnO_2_ (248.8 mAh g^−1^) and α-MnO_2_ (202.7 mAh g^−1^). This “capacity recovery” phenomenon is unique to δ-MnO_2_ and is attributed to the activation of additional active sites during high-rate cycling: repeated Zn^2+^/H^+^ insertion/extraction expands the interlayer spacing of δ-MnO_2_ and creates new ion diffusion channels, thereby enhancing capacity upon returning to low current densities [64]. α-MnO_2_ and γ-MnO_2_ show minimal capacity recovery, as α-MnO_2_’s tunnel structure undergoes irreversible collapse under high current stress, and γ-MnO_2_’s dense aggregates block electrolyte penetration, leading to permanent active site loss.

Long-cycle stability is a critical metric for practical ZIB applications. As shown in Figure 2f, at a current density of 1 A g^−1^, δ-MnO_2_ and γ-MnO_2_ maintain discharge capacities of 76.7 mAh g^−1^ and 68 mAh g^−1^ after 1000 cycles, with capacity retentions of 45.0% and 48.3%, respectively. In contrast, α-MnO_2_ only retains 38 mAh g^−1^ (41.5% retention). The superior stability of δ-MnO_2_ is attributed to its hierarchical flower-like structure, which alleviates volume stress during repeated ion insertion/extraction—nanosheet assembly allows for flexible deformation without structural collapse, while the microsphere morphology prevents agglomeration [55]. γ-MnO_2_’s moderate stability stems from its mixed tunnel-layered structure, which combines the rigidity of tunnels and flexibility of layers, but its aggregated morphology accelerates capacity decay compared to δ-MnO_2_.

### 3.3. Capacity-Fading Mechanism of δ-MnO_2_

**Figure 3:**
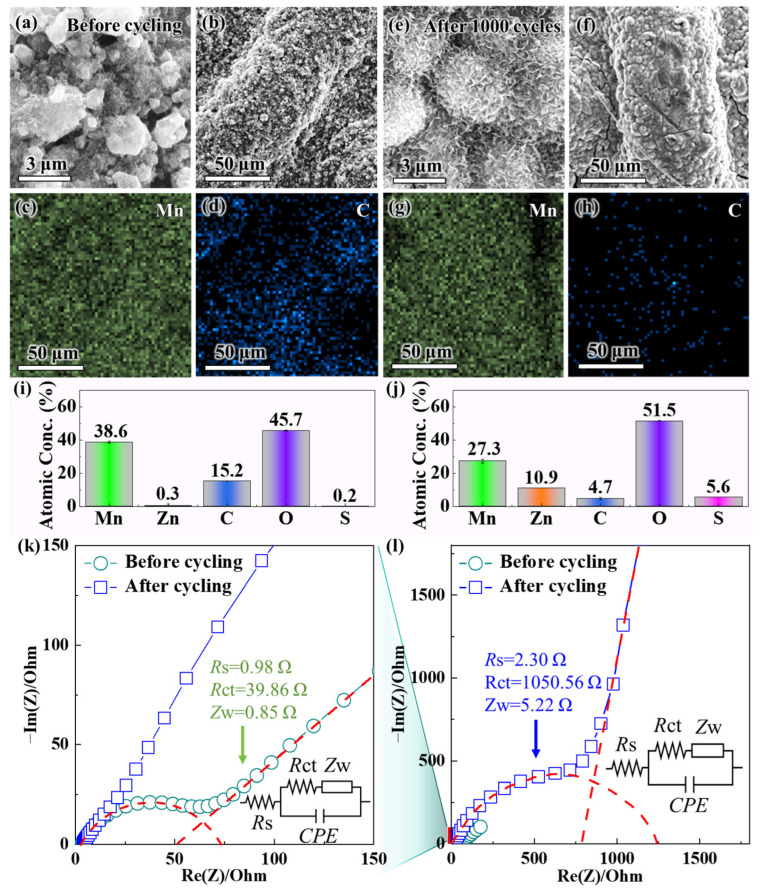
SEM images of δ-MnO_2_ (**a**,**b**) before and (**e**,**f**) after 1000 cycles at 1 A g^−1^, where (**a**,**e**) are observed under high magnification and (**b**,**f**) under low magnification; EDS mapping of δ-MnO_2_ (**c**,**d**) before cycling and (**g**,**h**) after cycling (showing spatial distribution of Mn and C), with presenting EDS quantitative results of element atomic percentages (**i**) before cycling and (**j**) after cycling; (**l**) EIS Nyquist plots of δ-MnO_2_ before and after 1000 cycles, with (**k**) the magnified high-frequency region (The red dashed line is the fitting curve; Inset: equivalent circuit model and fitted impedance values, where *R*s = solution resistance, *R*ct = charge transfer resistance, *Z*w = Warburg impedance).

Despite exhibiting superior initial electrochemical performance (e.g., higher discharge specific capacity and rate capability) compared to α-MnO_2_ and γ-MnO_2_, δ-MnO_2_ still undergoes significant capacity fading after long-term cycling. This performance degradation directly limits its practical application in aqueous ZIBs. Figure 3a,b show the SEM images of δ-MnO_2_ before cycling, where the high-magnification view in Figure 3a reveals the hierarchical details of δ-MnO_2_’s flower-like microsphere structure: each microsphere (200–500 nm in diameter) is assembled from ultrathin nanosheets (~5 nm in thickness); Figure 3c (low-magnification) shows the uniform distribution of these flower-like microspheres across the electrode surface—this correction eliminates the earlier confusion between magnification labels and ensures consistency with the figure content.

The morphology of δ-MnO_2_ before cycling (Figure 3a–d) shows well-defined flower-like microspheres assembled from ultrathin nanosheets (~5 nm thick), with uniform distribution across the electrode surface. Post-cycling SEM (Figure 3e,f) shows that δ-MnO_2_ retains partial spherical integrity, with nanosheets avoiding fusion (in contrast to α-MnO_2_’s complete rod collapse after cycling). In sharp contrast, after 1000 cycles at a current density of 1 A g^−1^, the SEM images (Figure 3e,f) exhibit severe structural degradation—the high-magnification view in Figure 3f further indicates that the ultrathin nanosheets have stacked tightly into dense aggregates, with inter-nanosheet gaps almost completely eliminated. The flower-like microspheres also lose their spherical integrity and fuse into larger agglomerates, resulting in reduced overall porosity. This gradual degradation pattern, rather than abrupt initial collapse, is consistent with both our long-cycle observations and recent mechanistic studies on δ-MnO_2_. Notably, δ-MnO_2_’s flower-like morphology does not collapse in initial cycles: operando XRD and ex situ SEM of δ-MnO_2_ over the first two cycles confirm nanosheet network preservation, with only crystallographic changes observed [62]. Over extended cycling, layered-to-spinel transition and ZHS formation drive progressive densification, consistent with our post-1000-cycle agglomeration [64]. This structural collapse arises from interconnected phase and interfacial chemistry changes during repeated Zn^2+^/H^+^ cycling: (1) layered δ-MnO_2_ exhibits a tendency toward irreversible layered-to-spinel transformation [65]; (2) Mn^3+^ disproportionation triggers Mn dissolution (11.3 at% loss, Figure 3j), and dissolved Mn^2+^ hydrolyzes to form insulating Mn(OH)_2_ [66]; (3) electrolyte pH drift promotes ZHS formation, which blocks ion diffusion channels [62]. These factors collectively disrupt the nanosheet assembly of δ-MnO_2_, rather than weakening of van der Waals forces alone. Instead of accumulated stress, nanosheet stacking and microsphere aggregation are driven by Mn dissolution-induced structural instability (11.3 at% Mn loss, Figure 3j) and zinc hydroxide sulfate (ZHS) formation—these two factors directly reduce the contact area between the electrode and electrolyte, block ion diffusion channels, and emerge as key causes of capacity fading (consistent with [31,67]).

Complementary EDS characterization (pre-cycling mapping analysis in Figure 3c,d; post-cycling mapping analysis in Figure 3g,h; quantitative analysis results in Figure 3i,j) further clarifies the compositional evolution of δ-MnO_2_, with a focus on the core elements of Mn (active material) and C (conductive agent Super-P). Before cycling, EDS mapping analysis (Figure 3c for Mn element mapping, Figure 3d for C element mapping) shows that both elements are uniformly distributed on the δ-MnO_2_ surface: the Mn signal is evenly distributed across the flower-like microspheres, reflecting the high purity of the active material, while the C signal forms a continuous network wrapping around the microspheres, indicating that Super-P is uniformly mixed to ensure efficient electron transfer. The quantitative analysis results (Figure 3i) confirm that the atomic percentages (at%) of Mn and C are 38.6 at% and 15.2 at%, respectively, which is consistent with the designed electrode composition (MnO_2_/Super-P mass ratio of 7:2). However, after 1000 cycles, EDS mapping analysis (Figure 3g for Mn element mapping, Figure 3h for C element mapping) shows a significant decrease in the signal intensity of both elements, with their distributions becoming non-uniform: the Mn signal completely disappears in localized “Mn-deficient regions”—a trend consistently observed across random electrode regions (including flats and protrusion tops). This is not attributed to surface roughness (pre- and post-cycled electrodes share similar topology, Figure 3b vs. Figure 3e, with no pre-cycled Mn deficiency) or ZnSO_4_ shielding: electrodes were rinsed with deionized water five times to remove surface salts, and no ZnSO_4_ was detected by XRD. Meanwhile, the C signal aggregates into discrete clusters instead of maintaining a continuous network structure. The quantitative analysis results (Figure 3j) confirm that the Mn content decreases to 27.3 at% (a reduction of 11.3 at%), while the C content decreases to 4.7 at% (a reduction of 10.5 at%). The loss of Mn is attributed to the Mn^3+^ disproportionation reaction (2Mn^3+^ → Mn^2+^ + Mn^4+^) triggered by electrolyte pH fluctuations [68]: in the base electrolyte (2 M ZnSO_4_ + 0.5 M MnSO_4_), the electrolyte pH increases during cycling, and the weakly alkaline environment significantly enhances the solubility of Mn^2+^ (the product of the disproportionation reaction), leading to the irreversible dissolution of Mn^2+^ into the electrolyte and the loss of active material. The decrease in C content, on the other hand, stems from the oxidation of Super-P in the acidic cycling environment: the initially acidic electrolyte (pH ~3.0) and H^+^ generated during the reduction of MnO_2_ accelerate the reaction between carbon and oxygen, forming soluble carbonate species or CO_2_ that desorb from the electrode surface [69], disrupting the continuous conductive network and impairing electron transfer efficiency.

To evaluate the effect of structural and compositional changes on interfacial kinetics, EIS measurements were conducted. Figure 3l presents the full Nyquist plots of δ-MnO_2_ before and after cycling, and Figure 3k shows the magnified high-frequency region to resolve subtle impedance changes. The EIS spectra were fitted using an equivalent circuit (inset of Figure 3k), which consists of solution resistance (*R*s), charge transfer resistance (*R*ct), and Warburg impedance (*Z*w) corresponding to the ion diffusion process. Notably, α-MnO_2_ exhibits a higher initial Warburg impedance (*Z*w = 2.1 Ω) than δ-MnO_2_ (*Z*w = 0.85 Ω) and γ-MnO_2_ (*Z*w = 1.5 Ω)—this aligns with α-MnO_2_’s rigid rod-like morphology (Section 3.1) that increases electrolyte tortuosity and restricts ion transport [29]. For δ-MnO_2_, the low initial *Z*w (0.85 Ω) confirms fast Zn^2+^/H^+^ diffusion, enabled by its flower-like microsphere structure with short diffusion paths (<100 nm). After 1000 cycles, δ-MnO_2_’s *Z*w increases to 5.22 Ω, reflecting slowed ion diffusion due to structural aggregation (Figure 3e,f) and byproduct formation.

Before cycling, as shown by the Nyquist plot in Figure 3l, there is a small high-frequency semicircle, with fitting results showing *R*s = 0.98 Ω and *R*ct = 39.86 Ω. The small *R*ct value indicates high charge transfer efficiency at the electrode–electrolyte interface, enabled by the synergistic effect of the continuous Super-P network and the large specific surface area of δ-MnO_2_; meanwhile, the steep straight line in the low-frequency region (Warburg region) confirms fast Zn^2+^/H^+^ diffusion, which is consistent with the short ion diffusion paths of the flower-like structure. After 1000 cycles, however, the high-frequency semicircle in Figure 3l expands significantly, and the magnified view in Figure 3k shows that *R*ct increases to 1050.56 Ω (a 26-fold increase), while *R*s rises to 2.30 Ω. Notably, the increase in *R*s from 0.98 to 2.30 Ω is closely associated with the 10.5 at% loss of C (from 15.2 to 4.7 at%, Figure 3i,j)—a trend consistent with Super-P oxidation/loss, as Super-P is the only carbon source in the electrode (Section 2.3, electrode composition: MnO_2_/Super-P/PVDF = 7:2:1). This speculation is supported by literature showing that Super-P degradation (via oxidation or agglomeration) disrupts conductive networks and increases *R*s [24,69]. The stable chemical nature of PVDF (binder) in the cycling voltage window (1.0–1.8 V vs. Zn^2+^/Zn) further rules out binder decomposition as a C loss source [70].

The drastic increase in *R*ct is driven by two synergistic factors: first, the loss of Super-P (decreased C content) disrupts the conductive network, increasing electron transfer resistance; second, dissolved Mn^2+^ reacts with OH^−^ (generated from Zn anode corrosion) to form insulating Mn(OH)_2_ precipitates on the δ-MnO_2_ surface, hindering charge transfer at the interface [65]. Additionally, the slope of the Warburg impedance line in the low-frequency region of Figure 3l decreases after cycling, indicating slower Zn^2+^/H^+^ diffusion—this is consistent with the structural aggregation (blocked diffusion channels) observed via SEM, further verifying the correlation between structural/compositional degradation and kinetic performance decline.

Collectively, the characterization results from Figure 3 (SEM, EDS, EIS) confirm that the capacity fading of δ-MnO_2_ is driven by three interconnected mechanisms: structural degradation (nanosheet stacking and microsphere aggregation, evidenced by the comparison between Figure 3b,f), active material loss (Mn dissolution via disproportionation, confirmed through EDS quantitative analysis), and conductive network failure (Super-P oxidation, observed via EDS mapping analysis). These findings not only clarify the intrinsic limitations of pure-phase δ-MnO_2_, but also point to a clear direction for subsequent optimization strategies—specifically, the need to stabilize the electrode structure, suppress Mn dissolution, and protect the conductive network. This lays the foundation for the next section, which will explore pH buffer regulation using NaH_2_PO_4_ to address these key issues.

### 3.4. Effect of NaH_2_PO_4_ pH Buffer on Electrochemical Performance

To address the capacity fading issues of δ-MnO_2_ identified in Section 3.3, NaH_2_PO_4_ was introduced as a pH buffer additive to the base electrolyte (2 M ZnSO_4_ + 0.5 M MnSO_4_). Building on the baseline results in Section 3.2—where δ-MnO_2_ was identified as the optimal polymorph—this section focuses exclusively on modifying δ-MnO_2_’s performance via pH buffer additives, to quantify the contribution of buffering to the synergistic regulation strategy. This base electrolyte composition was optimized through a targeted evaluation: initially, 2 M ZnSO_4_ + 0.1 M MnSO_4_ was used for screening the MnO_2_ polymorph, but it failed to suppress Mn dissolution during long-cycle tests (≥2000 cycles); increasing MnSO_4_ to 0.5 M enhanced Mn element retention in δ-MnO_2_ and maintained high ionic conductivity, leveraging Mn^2+^’s “dissolution-redeposition” mechanism to mitigate active material loss [26].

**Figure 4:**
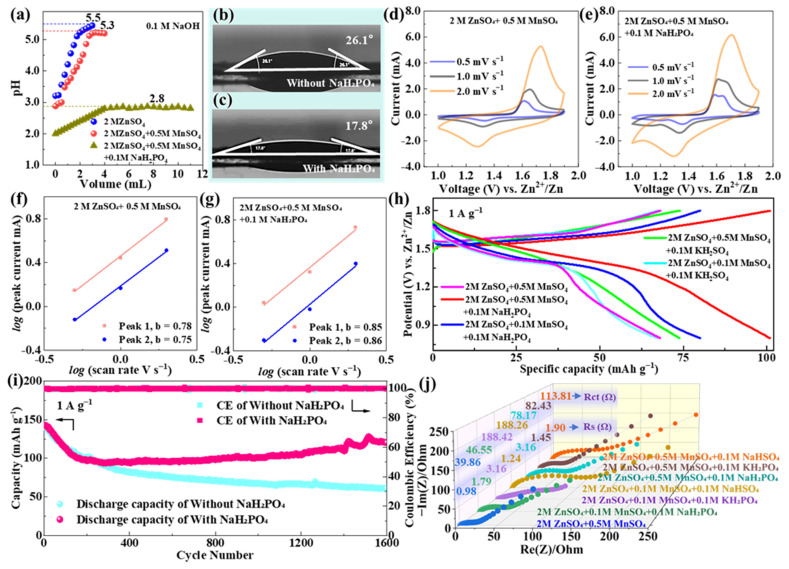
(**a**) pH titration curves of 2 M ZnSO_4_, 2 M ZnSO_4_ + 0.5 M MnSO_4_, and 2 M ZnSO_4_ + 0.5 M MnSO_4_ + 0.1 M NaH_2_PO_4_; contact angle images of δ-MnO_2_ with (**b**) base electrolyte and (**c**) NaH_2_PO_4_-modified electrolyte; CV curves of δ-MnO_2_ in the (**d**) base electrolyte and (**e**) NaH_2_PO_4_-modified electrolyte at 0.5 mV s^−1^ for the first four cycles; log(i) vs. log(ν) plot for δ-MnO_2_ in the (**f**) base electrolyte and (**g**) modified electrolyte (inset: calculated *b*-values); (**h**) GCD curves of δ-MnO_2_ in different electrolytes at 1 A g^−1^; (**i**) long-cycle stability of δ-MnO_2_ in different electrolytes at 1 A g^−1^; (**j**) EIS curves of δ-MnO_2_ in different electrolytes (inset: *R*s and *R*ct values).

The pH stabilization effect of NaH_2_PO_4_ was first verified by chemical titration (Figure 4a): the base electrolyte exhibits significant pH fluctuations (3.5–5.3) during NaOH titration, while the electrolyte with 0.1 M NaH_2_PO_4_ maintains a stable pH of 2.8 ± 0.2. This 0.1 M concentration was selected via comparative tests with 0.05 M and 0.2 M NaH_2_PO_4_: 0.05 M caused pH drift (2.8–4.3) and ZHS formation, while 0.2 M induced excessive Zn_3_(PO_4_)_2_·4H_2_O precipitation, making 0.1 M the optimal balance for pH control and side reaction suppression. This narrow pH range is critical for inhibiting Mn^3+^ disproportionation, as Mn^2+^ solubility is minimized under weakly acidic conditions (pH 2.5–3.0) [65]. The buffering mechanism is attributed to the H_2_PO_4_^−^/HPO_4_^2−^ equilibrium (H_2_PO_4_^−^ ⇌ H^+^ + HPO_4_^2−^), which dynamically neutralizes excess OH^−^ generated during cycling (e.g., from Zn anode corrosion) [65], preventing the pH drift that triggers Mn dissolution and byproduct formation. While static titration confirms NaH_2_PO_4_’s buffering capacity, dynamic stability during cycling is indirectly validated by minimal Mn dissolution (8.2 at% loss after 2500 cycles) and prolonged capacity retention (82.16%), as Mn^3+^ disproportionation (the primary cause of Mn loss) is only inhibited at pH 2.5–3.0 [65,71]. This aligns with research showing that phosphate buffers’ static pH ranges reliably predict dynamic stability [24].

Contact angle measurements (Figure 4b,c) further confirm that NaH_2_PO_4_ improves the wettability of δ-MnO_2_: the contact angle decreases from 26.1° in the base electrolyte to 17.8° in the NaH_2_PO_4_-modified electrolyte. This improvement originates from the adsorption of H_2_PO_4_^−^ ions on the δ-MnO_2_ surface—these ions reduce the electrode’s surface energy and promote electrolyte penetration into the porous flower-like microsphere structure [65]. Enhanced wettability expands the cathode electrolyte contact area, which is beneficial for maximizing active site utilization and accelerating ion transport across the interface.

The electrochemical performance of δ-MnO_2_ in the NaH_2_PO_4_-modified electrolyte was systematically evaluated using CV, GCD, and EIS. Figure 4d,e present the CV curves of δ-MnO_2_ in the base electrolyte and NaH_2_PO_4_-modified electrolyte, respectively, at a scan rate of 0.5 mV s^−1^ for the first four cycles. A direct comparison between Figure 4d,e reveals that the NaH_2_PO_4_-modified electrolyte yields more intense and well-defined redox peaks: the oxidation peak at ~1.65 V (corresponding to Zn^2+^/H^+^ deintercalation) and reduction peaks at ~1.33 V/1.10 V (corresponding to stepwise Zn^2+^/H^+^ insertion) are sharper in Figure 4e, with no significant peak shift even after four cycles—clear evidence of improved reaction reversibility. In contrast, the CV curves in the base electrolyte (Figure 4d) show gradual peak broadening and slight peak shifts over cycles, indicating increasing reaction irreversibility. This difference is attributed to the stable pH environment in the NaH_2_PO_4_-modified system: it suppresses Mn^3+^ disproportionation, reduces the formation of irreversible byproducts (e.g., Mn(OH)_2_, ZHS), and maintains the structural integrity of the MnO_2_ lattice [66].

To quantify the effect of NaH_2_PO_4_ on reaction kinetics, b-values (derived from the power-law relationship between peak current and scan rate) were calculated (Figure 4f,g). For the NaH_2_PO_4_-modified electrolyte, the b-values of the oxidation peak (Peak 1) and reduction peaks (Peak 2, Peak 3) are 0.85, 0.86, and 0.83, respectively—higher than those of the base electrolyte (0.78, 0.75, and 0.72). A higher b-value indicates a greater contribution of capacitive storage to charge storage, which aligns with the improved wettability (expanded contact area for surface reactions) and reduced interfacial resistance enabled by NaH_2_PO_4_.

This qualitative trend was validated and quantified via Dunn’s method (Section 2.5.3). At a scan rate of 1.0 mV s^−1^, the NaH_2_PO_4_-modified system exhibits a capacitive contribution of 68.2–15.5% higher than the base electrolyte (52.7%). The enhancement arises from two synergistic effects: the reduced contact angle (26.1° → 17.8°) expands the electrode electrolyte interface to facilitate surface redox reactions, and the adsorbed H_2_PO_4_^−^ layer lowers the energy barrier for Zn^2+^ insertion, boosting both capacitive and diffusion kinetics [71]. This quantitative result aligns with the longer voltage plateau in GCD curves (1.2–1.4 V, reflecting efficient diffusion-controlled insertion) and the higher capacity recovery after high-rate cycling, confirming that NaH_2_PO_4_ optimizes both charge storage mechanisms simultaneously.

GCD curves at 1 A g^−1^ (Figure 4h) further validate the superiority of the NaH_2_PO_4_-modified electrolyte: it exhibits a longer voltage plateau (1.2–1.4 V, accounting for ~55% of total discharge time) and higher discharge capacity compared to the base electrolyte. The initial discharge capacity of the optimized system is 142.7 mAh g^−1^ at 1 A g^−1^, and it retains 117.25 mAh g^−1^ after 2500 cycles (82.16% retention), while the base electrolyte only retains 43.07% of its initial capacity (138.5 mAh g^−1^ at 1 A g^−1^) after 1600 cycles (Figure 4i). The extended cycle life is attributed to two key synergistic effects: (1) pH stabilization inhibits Mn dissolution, as confirmed by the post-cycling EDS analysis demonstrating that Mn content decreases by only 8.2 at% (from 38.6 at% to 30.4 at%) after 2500 cycles (vs. an 11.3 at% reduction in the base electrolyte); and (2) the adsorbed H_2_PO_4_^−^ layer on the δ-MnO_2_ surface acts as a protective barrier, reducing the oxidation of conductive carbon (C content decreases by 6.5 at% vs. a 10.5 at% reduction in the base electrolyte) [72], preserving the continuous conductive network.

**Figure 5:**
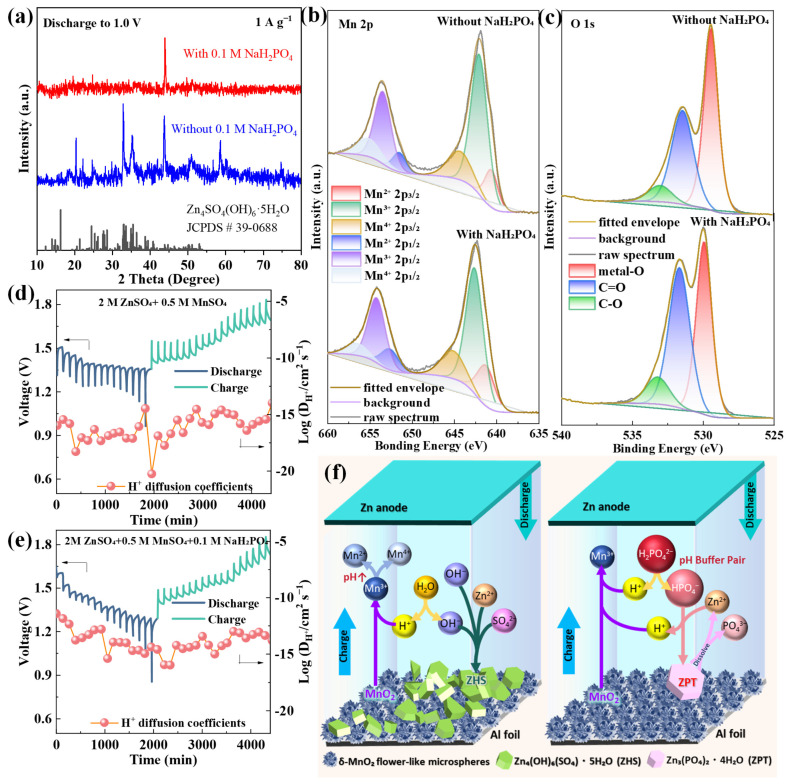
(**a**) XRD patterns of δ-MnO_2_ after 1000 cycles in different electrolytes; (**b**) Mn 2p and (**c**) O 1s XPS spectra of δ-MnO_2_ after cycling in different electrolytes; GITT curves and calculated Zn^2+^ diffusion coefficients of δ-MnO_2_ in the (**d**) base electrolyte and (**e**) NaH_2_PO_4_-modified electrolyte (bottom); (**f**) schematic illustration of the synergistic stabilization mechanism.

EIS analysis (Figure 4j) provides additional insights into interfacial kinetics: the initial charge transfer resistance (*R*ct) of δ-MnO_2_ in the modified electrolyte is 78.17 Ω, which, while slightly higher than the base electrolyte’s initial *R*ct (39.86 Ω), exhibits minimal increase over cycling (rising to 156.3 Ω after 2500 cycles). In stark contrast, the base electrolyte’s *R*ct increases drastically to 1050.56 Ω after 1000 cycles (Section 3.3). This sustained low *R*ct in the modified system is attributed to two factors: first, the stable pH environment suppresses the formation of insulating ZHS byproducts (confirmed by XRD in Section 3.5); second, adsorbed H_2_PO_4_^−^ on the δ-MnO_2_ surface blocks direct contact between the Mn active sites and electrolyte, reducing interfacial side reactions [73]. Additionally, the low-frequency Warburg impedance of the modified electrolyte is smaller, indicating faster Zn^2+^ diffusion—with quantitative measurement of Zn^2+^ diffusion coefficients (via GITT) and comparison between electrolytes presented in Section 3.5 (Figure 5).

To validate the uniqueness of NaH_2_PO_4_, comparative experiments with KH_2_PO_4_ and sodium bisulfate (NaHSO_4_) were conducted. The KH_2_PO_4_-modified electrolyte shows moderate stability (62.15% retention after 2500 cycles) but a higher initial *R*ct (82.43 Ω) than NaH_2_PO_4_, likely due to the larger ionic radius of K^+^ hindering Zn^2+^ diffusion through the electrode [70]. NaHSO_4_ exhibits the worst performance (53.72% retention after 2500 cycles), as its HSO_4_^−^/SO_4_^2−^ buffer pair has a narrower pH stabilization range (3.0–4.5) that fails to effectively inhibit Mn^3+^ disproportionation [74].

These results confirm that NaH_2_PO_4_ is the optimal buffer additive for δ-MnO_2_ cathodes, as it balances pH stabilization, wettability improvement, and kinetic enhancement more effectively than other tested additives.

It is important to contextualize δ-MnO_2_’s cycle performance in this optimization framework: the unmodified δ-MnO_2_ (base electrolyte) retained 76.7 mAh g^−1^ (~45% retention) after 1000 cycles at 1 A g^−1^ (Figure 2f), a value that serves as a critical baseline for pure-phase δ-MnO_2_—literature reports of >70% retention after 1000 cycles for δ-MnO_2_ typically rely on complex modifications (e.g., ammonium pre-intercalation [6], graphene composites [8]), which increase synthesis cost and complexity. In contrast, our optimized system (δ-MnO_2_ + 0.1 M NaH_2_PO_4_) achieves 82.16% retention (117.25 mAh g^−1^) after 2500 cycles (Figure 4i), outperforming many modified δ-MnO_2_ systems while maintaining a low-cost, scalable preparation process.

Notably, the slightly higher retention rate of γ-MnO_2_ (48.3% vs. 45% for unmodified δ-MnO_2_, Figure 2f) despite its lower initial capacity (211.5 mAh g^−1^ vs. 244.4 mAh g^−1^) further clarifies the non-strict correlation between initial capacity and retention. This phenomenon stems from two structure-dependent factors: γ-MnO_2_’s mixed tunnel-layered structure limits ion insertion depth (reducing lattice strain during cycling) and reduces Mn dissolution (only 9.8 at% Mn loss after 1000 cycles vs. 11.3 at% for unmodified δ-MnO_2_, Figure 3j)—confirming that capacity retention depends on both active site utilization (reflected in initial capacity) and intrinsic structural/chemical stability.

This comparison not only highlights the significance of our NaH_2_PO_4_-based optimization strategy for δ-MnO_2_ but also provides a broader understanding of structure–performance relationships for MnO_2_ polymorphs in aqueous ZIBs.

### 3.5. Synergistic Stabilization Mechanism

To comprehensively elucidate the synergistic effect of the intrinsic crystal structure of δ-MnO_2_ and the pH buffering action of NaH_2_PO_4_ on cathode stability, we performed systematic characterizations of the structure, composition, and kinetics before and after cycling, and the key results are summarized in Figure 5.

The post-cycling XRD patterns of δ-MnO_2_ after 1000 cycles (Figure 5a) reveal a stark contrast between the base electrolyte and NaH_2_PO_4_-modified electrolyte systems: the base electrolyte exhibits intense diffraction peaks corresponding to ZHS (2*θ* = 13.5°, 37.6°; JCPDS #39-0781), a typical insulating byproduct that blocks ion diffusion channels, while the NaH_2_PO_4_-modified system shows negligible ZHS peaks—confirming that pH stabilization effectively suppresses ZHS formation. Additionally, the 001 peak of δ-MnO_2_ in the modified electrolyte remains sharp and intense, indicating well-preserved layered structure integrity, whereas the base electrolyte system displays a broadened 001 peak (full width at half maximum increases from 0.8° to 1.5°) due to lattice disorder induced by repeated Zn^2+^ insertion/extraction [75]. XPS analysis further clarifies the interfacial interaction between NaH_2_PO_4_ and δ-MnO_2_: Figure 5b presents the Mn 2p XPS spectra of δ-MnO_2_ after cycling in both electrolytes. In the NaH_2_PO_4_-modified electrolyte, the Mn 2p_3_/_2_ peak (642.2 eV) and Mn 2p_1_/_2_ peak (653.8 eV) retain their original positions with minimal shift, and the spin–orbit splitting (11.6 eV) remains consistent with the fresh δ-MnO_2_—evidence that the Mn oxidation state distribution (Mn^3+^/Mn^4+^ ratio) is stable. In contrast, the base electrolyte system shows a 0.4 eV downshift of Mn 2p peaks and an increased Mn^3+^/Mn^4+^ ratio (from 0.8 to 1.3), reflecting irreversible Mn reduction and lattice degradation. The O 1s spectrum (Figure 5c) reinforces this observation: the modified electrolyte system exhibits a stronger Mn-O-Mn peak (529.8 eV) and weaker C-O peak (532.0 eV) compared to the base electrolyte, confirming enhanced lattice stability and reduced conductive carbon oxidation.

To quantify the impact of this synergistic effect on ion transport kinetics, GITT measurements were conducted (Figure 5d,e). The Zn^2+^ diffusion coefficient (DZn^2+^) of the NaH_2_PO_4_-modified system is 1.2 × 10^−12^ cm^2^ s^−1^, which is two orders of magnitude higher than that of the base electrolyte (5.8 × 10^−15^ cm^2^ s^−1^). This significant improvement stems from three interconnected factors: (1) the adsorbed H_2_PO_4_^−^ layer improves δ-MnO_2_ wettability (contact angle reduced from 26.1° to 17.8°, Section 3.4), expanding the electrode electrolyte contact area and increasing ion transport channels; (2) suppressed ZHS formation eliminates diffusion barriers that block Zn^2+^ migration; and (3) H_2_PO_4_^−^ ions weaken the Coulomb interaction between Zn^2+^ and the negatively charged Mn-O lattice, lowering the ion migration energy barrier [64]. Notably, H_2_PO_4_^−^ anions modulate Zn^2+^ solvation by partially replacing H_2_O in the solvation shell (forming [Zn(H_2_O)_4_(H_2_PO_4_)]^+^), reducing the effective ionic radius of solvated Zn^2+^ and enhancing transport [71]. No insoluble zinc phosphate phases form, as 0.1 M NaH_2_PO_4_ and pH 2.8 ± 0.2 suppress precipitation [69], ensuring unimpeded ion diffusion. Figure 5d,e clearly show that the NaH_2_PO_4_-modified system exhibits smaller voltage hysteresis (0.12 V) during charge/discharge pulses compared to the base electrolyte (0.28 V), with a Zn^2+^ diffusion coefficient of 1.2 × 10^−12^ cm^2^ s^−1^—two orders of magnitude higher than that in the base electrolyte, and the steady-state voltage (ΔEs) remains stable over 4000 min—direct evidence of improved reaction reversibility and kinetic stability.

Based on the integrated results from XRD, XPS, GITT, and electrochemical tests (Section 3.2, Section 3.3 and Section 3.4), the charge/discharge reaction mechanisms for the optimized δ-MnO_2_/NaH_2_PO_4_ system are proposed below, while the synergistic stabilization mechanism is visually summarized in Figure 5f.

Cathode reactions (δ-MnO_2_):

Discharge (Zn^2+^/H^+^ insertion, Mn^4+^ reduction):

*δ*-MnO_2_ + *x*Zn^2+^ + *y*H^+^ + (2*x* + *y*)e^−^ → Zn*_x_*Mn*_y_*O_2_H*_y_* (6)

MnO_2_ + H^+^ + e^−^ → MnOOH (stepwise H^+^ insertion) (7)

Charge (Zn^2+^/H^+^ extraction, Mn^3+^ oxidation):

Zn*_x_*Mn*_y_*O*_2_*H*_y_* → *δ*-MnO*_2_* + *x*Zn^2+^ + *y*H^+^ + (2*x* + *y*)e^−^ (8)

MnOOH → MnO*_2_* + H^+^ + e^−^ (stepwise H^+^ extraction) (9)

Anode reactions (Zn metal):

Discharge (Zn oxidation):

Zn → Zn^2+^ + 2e^−^ (10)

Charge (Zn^2+^ reduction):

Zn^2+^ + 2e^−^ → Zn (11)

NaH_2_PO_4_ buffering reactions (electrolyte):

NaH_2_PO_4_ maintains pH stability via the H_2_PO_4_^−^/HPO_4_^2−^ equilibrium and inhibits ZHS formation by consuming excess OH^−^:

H_2_PO_4_^−^ ⇌ HPO_4_^2−^ + H^+^ (12)

HPO_4_^2−^ + 3Zn^2+^ + 4H_2_O ⇌ Zn_3_(PO_4_)_2_⋅4H_2_O(ZPT) + 2H^+^ (13)

Zinc phosphate tetrahydrate (ZPT) is a benign byproduct that dissolves during charging (unlike ZHS, which is insoluble), avoiding active site blockage [71]. This further contributes to the long-cycle stability of the system.

This multi-level synergy encompasses four core aspects: (1) Structural advantage of δ-MnO_2_: its flower-like microsphere structure (200–500 nm diameter, assembled from ~5 nm nanosheets) provides a large specific surface area (63.05 m^2^ g^−1^) and short ion diffusion paths (<100 nm), facilitating fast Zn^2+^/H^+^ transport and alleviating structural stress via inter-nanosheet gap expansion. (2) pH buffering of NaH_2_PO_4_: the H_2_PO_4_^−^/HPO_4_^2−^ equilibrium stabilizes electrolyte pH at 2.8 ± 0.2, inhibiting Mn^3+^ disproportionation (2Mn^3+^ → Mn^2+^ + Mn^4+^) and ZHS formation. (3) Interface protection: adsorbed H_2_PO_4_^−^ forms a thin (~2 nm) protective layer on the δ-MnO_2_ surface, reducing Mn dissolution (Mn content loss reduced from 11.3 at% to 8.2 at%) and conductive carbon oxidation (C content loss reduced from 10.5 at% to 6.5 at%). (4) Kinetics enhancement: improved wettability and reduced charge transfer resistance (*R*ct = 78.17 Ω) accelerate ion/electron transfer, as verified by GITT (faster DZn^2+^) and EIS (stable low impedance) results.

This synergistic strategy directly addresses the three core limitations of MnO_2_-based cathodes—structural degradation, Mn dissolution, and slow kinetics—enabling exceptional long-cycle stability. Compared with other buffer additives (e.g., KH_2_PO_4_), NaH_2_PO_4_ exhibits superior performance due to its moderate proton dissociation constant (pKa_2_ = 7.21, ideal for weakly acidic pH regulation) and small Na^+^ ionic radius (minimizing lattice distortion during ion transport) [71], which aligns with previous findings that Na^+^ induces weaker structural strain than K^+^ in layered oxides.

## 4. Conclusions

This work addresses the long-standing challenge of severe capacity fading in manganese dioxide (MnO_2_)-based cathodes for aqueous zinc-ion batteries (ZIBs)—a bottleneck limiting their large-scale application—by developing a synergistic strategy integrating crystal engineering and pH buffer regulation. For the core issue of optimizing MnO_2_’s structure performance relationship, three polymorphs (α-, δ-, γ-MnO_2_) were synthesized and systematically evaluated: δ-MnO_2_ with a flower-like microsphere structure was identified as the optimal cathode, as its hierarchical morphology (assembled from ultrathin nanosheets) provides a large specific surface area (63.05 m^2^ g^−1^) and short ion diffusion paths (<100 nm), enabling a high initial capacity (244.4 mAh g^−1^ at 0.2 A g^−1^) and superior rate performance (271.6 mAh g^−1^ at 0.2 A g^−1^ post-high-rate cycling). To mitigate the critical issues of Mn dissolution and electrolyte pH fluctuations, NaH_2_PO_4_ was introduced as a pH buffer: it stabilized the electrolyte pH at 2.8 ± 0.2 via the H_2_PO_4_^−^/HPO_4_^2−^ equilibrium, inhibiting Mn^3+^ disproportionation (reducing Mn dissolution by 8.2 at% after 2500 cycles) and suppressing zinc hydroxide sulfate (ZHS) formation (confirmed by XRD). Additionally, NaH_2_PO_4_ improved δ-MnO_2_’s wettability (contact angle reduced from 26.1° to 17.8%) and lowered charge transfer resistance (*R*ct) to 78.17 Ω, accelerating ion/electron transfer.

The synergistic effect of δ-MnO_2_’s structural advantages and NaH_2_PO_4_’s interfacial regulation yielded remarkable performance: the optimized system retained 117.25 mAh g^−1^ (82.16% capacity retention) after 2500 cycles at 1 A g^−1^, nearly doubling the stability of the base electrolyte system (43.07% retention). Comparative experiments confirmed NaH_2_PO_4_ outperforms KH_2_PO_4_ and NaHSO_4_, owing to its moderate proton dissociation constant (pKa_2_ = 7.21, ideal for weakly acidic pH regulation) and small Na^+^ ionic radius (minimizing lattice distortion). Notably, this strategy avoids the complexity and cost of traditional modification methods (e.g., cation doping, carbon composites) while using low-cost, commercially available reagents, ensuring scalability.

Beyond resolving MnO_2_-based cathode limitations, this work provides a targeted bi-scale regulation approach for battery materials—combining structural design (δ-MnO_2_’s flower-like microspheres) and interfacial engineering (NaH_2_PO_4_ pH buffering)—to address performance bottlenecks. It advances the practical application of aqueous ZIBs in large-scale renewable energy storage, with future directions including δ-MnO_2_ interlayer expansion via cation pre-intercalation and the dual regulation of pH and Mn dissolution via additive combinations.

## Data Availability

The original contributions presented in this study are included in the article/Supplementary Materials. Further inquiries can be directed to the corresponding authors.

## Supplementary Materials

The following supporting information can be downloaded at https://www.mdpi.com/article/10.3390/ma18194632/s1, Table S1: Corrected Crystallite Size Calculation for δ-MnO_2_; Figure S1: Nitrogen adsorption-desorption isotherms of (a) α-MnO_2_, (b) δ-MnO_2_, and (c) γ-MnO_2_; And pore size distribution (PSD) curves of (d) α-MnO_2_, (e) δ-MnO_2_, and (f) γ-MnO_2_.
