# Supplementary material for: Synergistic Regulation of δ-MnO2 Cathode via Crystal Engineering and pH Buffering for Long-Cycle Aqueous Zinc-Ion Batteries
Source: Materials (Basel). 2025 Oct 8;18(19):4632. doi: 10.3390/ma18194632 (PMC12526178; doi:10.3390/ma18194632)
Supplement: Supplementary file 1 [file materials-18-04632-s001.zip › materials-3883261-supplementary.pdf]

Supporting Information

Synergistic Regulation of δ-MnO2 Cathode via Crystal Engineering and pH Buffering for Long-Cycle Aqueous Zinc-Ion Batteries

Fan Zhang [1], Haotian Yu [1], Qiongyue Zhang [1], Yahao Wang [1], Haodong Ren [1], Huirong Liang [1], Jinrui Li [1], Yuanyuan Feng [1], Bin Zhao [2,*] and Xiaogang Han [2,*]

[1] School of Electric Power, Civil Engineering and Architecture, Shanxi University, Taiyuan 030006, China
[2] State Key Laboratory of Electrical Insulation and Power Equipment, School of Electrical Engineering, Xi'an Jiaotong University, Xi'an 710049, China
* Correspondence: zhaobin87@xjtu.edu.cn (B.Z.); xiaogang.han@xjtu.edu.cn (X.H.)

Table S1. Corrected Crystallite Size Calculation for δ-MnO_2.

Peak (hkl)	2θ (°)	$\beta_{measured}$ (°)	$\beta_{measured}$ (rad)	β_{instr} (rad)	$\beta_{corrected}$ (rad)	cos θ	D (nm)
(001)	12.2	0.80	0.01396	0.00523	0.01287	0.9945	9.8
(002)	24.7	0.92	0.01606	0.00551	0.01503	0.9093	10.5
(11-1)	36.8	1.05	0.01832	0.00610	0.01725	0.8018	10.1
(004)	65.0	1.23	0.02147	0.00701	0.02038	0.4226	10.4
Average	--	--	--	--	--	--	10.2±0.5

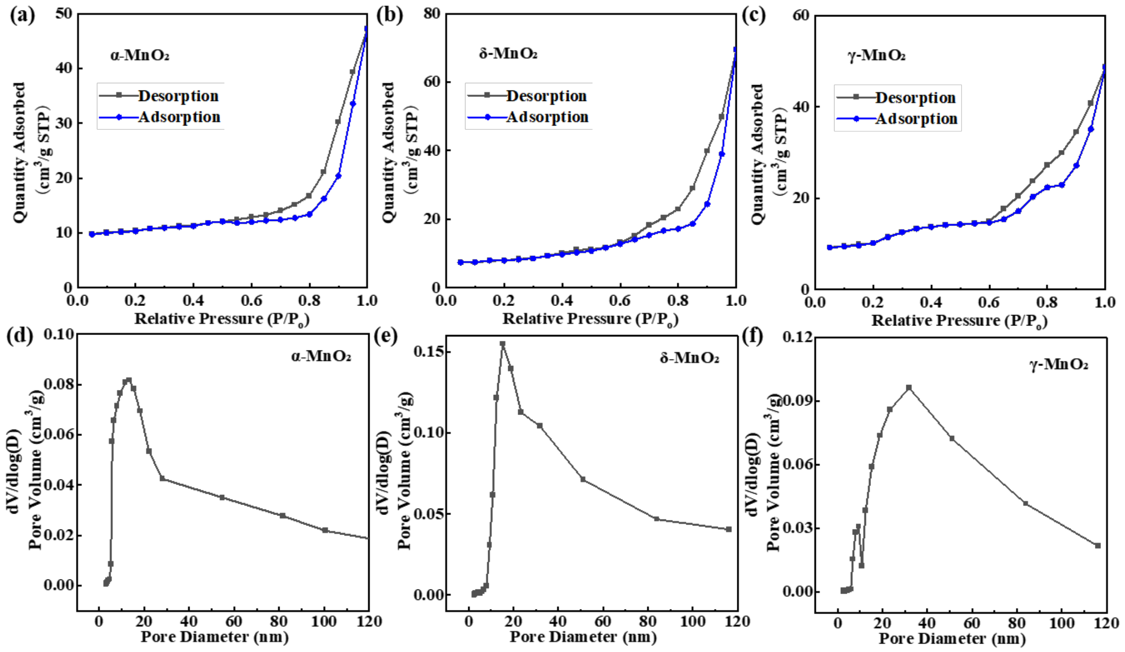

Figure S1. Nitrogen adsorption-desorption isotherms of (a) α-MnO_2, (b) δ-MnO_2, and (c) γ-MnO_2; And pore size distribution (PSD) curves of (d) α-MnO_2, (e) δ-MnO_2, and (f) γ-MnO_2.
